# THE CONCISE GUIDE TO PHARMACOLOGY 2019/20: Introduction and Other Protein Targets

**DOI:** 10.1111/bph.14747

**Published:** 2019-11-11

**Authors:** Stephen PH Alexander, Eamonn Kelly, Alistair Mathie, John A Peters, Emma L Veale, Jane F Armstrong, Elena Faccenda, Simon D Harding, Adam J Pawson, Joanna L Sharman, Christopher Southan, O Peter Buneman, John A Cidlowski, Arthur Christopoulos, Anthony P Davenport, Doriano Fabbro, Michael Spedding, Jörg Striessnig, Jamie A Davies, CGTP Collaborators, Thiruma V Arumugam, Andrew Bennett, Benita Sjögren, Christopher Sobey, Szu Shen Wong

**Affiliations:** ^1^ School of Life Sciences University of Nottingham Medical School Nottingham NG7 2UH UK; ^2^ School of Physiology, Pharmacology and Neuroscience University of Bristol Bristol BS8 1TD UK; ^3^ Medway School of Pharmacy The Universities of Greenwich and Kent at Medway Anson Building, Central Avenue, Chatham Maritime Chatham Kent ME4 4TB UK; ^4^ Neuroscience Division, Medical Education Institute, Ninewells Hospital and Medical School University of Dundee Dundee DD1 9SY UK; ^5^ Centre for Discovery Brain Sciences University of Edinburgh Edinburgh EH8 9XD UK; ^6^ Laboratory for Foundations of Computer Science, School of Informatics University of Edinburgh Edinburgh EH8 9LE UK; ^7^ National Institute of Environmental Health Sciences National Institutes of Health, Department of Health and Human Services Research Triangle Park NC 27709 USA; ^8^ Monash Institute of Pharmaceutical Sciences and Department of Pharmacology Monash University Parkville Victoria 3052 Australia; ^9^ Clinical Pharmacology Unit University of Cambridge Cambridge CB2 0QQ UK; ^10^ PIQUR Therapeutics Basel 4057 Switzerland; ^11^ Spedding Research Solutions SARL Le Vésinet 78110 France; ^12^ Pharmacology and Toxicology, Institute of Pharmacy University of Innsbruck A-6020 Innsbruck Austria; ^13^ Singapore Singapore; ^14^ Nottingham UK; ^15^ West Lafayette USA; ^16^ Clayton Australia; ^17^ Keele UK

## Abstract

The Concise Guide to PHARMACOLOGY 2019/20 is the fourth in this series of biennial publications. The Concise Guide provides concise overviews of the key properties of nearly 1800 human drug targets with an emphasis on selective pharmacology (where available), plus links to the open access knowledgebase source of drug targets and their ligands (http://www.guidetopharmacology.org/), which provides more detailed views of target and ligand properties. Although the Concise Guide represents approximately 400 pages, the material presented is substantially reduced compared to information and links presented on the website. It provides a permanent, citable, point‐in‐time record that will survive database updates. The full contents of this section can be found at http://onlinelibrary.wiley.com/doi/10.1111/bph.14747. In addition to this overview, in which are identified Other protein targets which fall outside of the subsequent categorisation, there are six areas of focus: G protein‐coupled receptors, ion channels, nuclear hormone receptors, catalytic receptors, enzymes and transporters. These are presented with nomenclature guidance and summary information on the best available pharmacological tools, alongside key references and suggestions for further reading. The landscape format of the Concise Guide is designed to facilitate comparison of related targets from material contemporary to mid‐2019, and supersedes data presented in the 2017/18, 2015/16 and 2013/14 Concise Guides and previous Guides to Receptors and Channels. It is produced in close conjunction with the International Union of Basic and Clinical Pharmacology Committee on Receptor Nomenclature and Drug Classification (NC‐IUPHAR), therefore, providing official IUPHAR classification and nomenclature for human drug targets, where appropriate.

1

### Table of contents

#### S1 Introduction and Other Protein Targets

S6 Adiponectin receptors
S7 Blood coagulation components
S8 Non‐enzymatic BRD containing proteins
S9 Carrier proteins
S9 CD molecules
S11 Methyllysine reader proteins
S11 Fatty acid‐binding proteins
S14 Notch receptors
S15 Regulators of G protein Signaling (RGS) proteins
S18 Sigma receptors
S19 Tubulins

#### S21 G protein‐coupled receptors

S23 Orphan and other 7TM receptors
S24 Class A Orphans
S26 Class C Orphans
S33 Taste 1 receptors
S34 Taste 2 receptors
S35 Other 7TM proteins
S36 5‐Hydroxytryptamine receptors
S39 Acetylcholine receptors (muscarinic)
S41 Adenosine receptors
S42 Adhesion Class GPCRs
S45 Adrenoceptors
S48 Angiotensin receptors
S50 Apelin receptor
S51 Bile acid receptor
S51 Bombesin receptors
S53 Bradykinin receptors
S54 Calcitonin receptors
S56 Calcium‐sensing receptor
S57 Cannabinoid receptors
S58 Chemerin receptors
S59 Chemokine receptors
S63 Cholecystokinin receptors
S64 Class Frizzled GPCRs
S67 Complement peptide receptors
S68 Corticotropin‐releasing factor receptors
S69 Dopamine receptors
S71 Endothelin receptors
S72 G protein‐coupled estrogen receptor
S73 Formylpeptide receptors
S74 Free fatty acid receptors
S76 GABA_B_ receptors
S78 Galanin receptors
S79 Ghrelin receptor
S80 Glucagon receptor family
S81 Glycoprotein hormone receptors
S82 Gonadotrophin‐releasing hormone receptors
S83 GPR18, GPR55 and GPR119
S84 Histamine receptors
S86 Hydroxycarboxylic acid receptors
S87 Kisspeptin receptor
S88 Leukotriene receptors
S89 Lysophospholipid (LPA) receptors
S90 Lysophospholipid (S1P) receptors
S92 Melanin‐concentrating hormone receptors
S93 Melanocortin receptors
S94 Melatonin receptors
S95 Metabotropic glutamate receptors
S97 Motilin receptor
S98 Neuromedin U receptors
S99 Neuropeptide FF/neuropeptide AF receptors
S100 Neuropeptide S receptor
S101 Neuropeptide W/neuropeptide B receptors
S102 Neuropeptide Y receptors
S103 Neurotensin receptors
S104 Opioid receptors
S106 Orexin receptors
S107 Oxoglutarate receptor
S108 P2Y receptors
S110 Parathyroid hormone receptors
S111 Platelet‐activating factor receptor
S112 Prokineticin receptors
S113 Prolactin‐releasing peptide receptor
S114 Prostanoid receptors
S116 Proteinase‐activated receptors
S117 QRFP receptor
S118 Relaxin family peptide receptors
S120 Somatostatin receptors
S121 Succinate receptor
S122 Tachykinin receptors
S123 Thyrotropin‐releasing hormone receptors
S124 Trace amine receptor
S125 Urotensin receptor
S126 Vasopressin and oxytocin receptors
S127 VIP and PACAP receptors

#### S142 Ion channels

S143 Ligand‐gated ion channels
S144 5‐HT_3_ receptors
S146 Acid‐sensing (proton‐gated) ion channels (ASICs)
S148 Epithelial sodium channel (ENaC)
S149 GABA_A_ receptors
S155 Glycine receptors
S158 Ionotropic glutamate receptors
S164 IP_3_ receptor
S165 Nicotinic acetylcholine receptors
S168 P2X receptors
S170 ZAC
S171 Voltage‐gated ion channels
S171 CatSper and Two‐Pore channels
S173 Cyclic nucleotide‐regulated channels
S175 Potassium channels
S175 Calcium‐ and sodium‐activated potassium channels
S178 Inwardly rectifying potassium channels
S182 Two P domain potassium channels
S185 Voltage‐gated potassium channels
S189 Ryanodine receptors
S190 Transient Receptor Potential channels
S204 Voltage‐gated calcium channels
S207 Voltage‐gated proton channel
S208 Voltage‐gated sodium channels
S210 Other ion channels
S210 Aquaporins
S212 Chloride channels
S213 ClC family
S215 CFTR
S216 Calcium activated chloride channel
S217 Maxi chloride channel
S218 Volume regulated chloride channels
S219 Connexins and Pannexins
S221 Piezo channels
S222 Sodium leak channel, non‐selective

#### S229 Nuclear hormone receptors

S230 1A. Thyroid hormone receptors
S231 1B. Retinoic acid receptors
S232 1C. Peroxisome proliferator‐activated receptors
S233 1D. Rev‐Erb receptors
S234 1F. Retinoic acid‐related orphans
S234 1H. Liver X receptor‐like receptors
S235 1I. Vitamin D receptor‐like receptors
S236 2A. Hepatocyte nuclear factor‐4 receptors
S237 2B. Retinoid X receptors
S238 2C. Testicular receptors
S238 2E. Tailless‐like receptors
S239 2F. COUP‐TF‐like receptors
S239 3B. Estrogen‐related receptors
S240 4A. Nerve growth factor IB‐like receptors
S241 5A. Fushi tarazu F1‐like receptors
S241 6A. Germ cell nuclear factor receptors
S242 0B. DAX‐like receptors
S242 Steroid hormone receptors
S243 3A. Estrogen receptors
S244 3C. 3‐Ketosteroid receptors

#### S247 Catalytic receptors

S248 Cytokine receptor family
S249 IL‐2 receptor family
S251 IL‐3 receptor family
S252 IL‐6 receptor family
S254 IL‐12 receptor family
S255 Prolactin receptor family
S256 Interferon receptor family
S257 IL‐10 receptor family
S258 Immunoglobulin‐like family of IL‐1 receptors
S259 IL‐17 receptor family
S259 GDNF receptor family
S260 Integrins
S264 Pattern recognition receptors
S264 Toll‐like receptor family
S266 NOD‐like receptor family
S268 RIG‐I‐like receptor family
S269 Receptor Guanylyl Cyclase (RGC) family
S269 Transmembrane quanylyl cyclases
S270 Nitric oxide (NO)‐sensitive (soluble) guanylyl cyclase
S271 Receptor tyrosine kinases (RTKs)
S272 Type I RTKs: ErbB (epidermal growth factor) receptor family
S273 Type II RTKs: Insulin receptor family
S274 Type III RTKs: PDGFR, CSFR, Kit, FLT3 receptor family
S275 Type IV RTKs: VEGF (vascular endothelial growth factor) receptor family
S275 Type V RTKs: FGF (broblast growth factor) receptor family
S276 Type VI RTKs: PTK7/CCK4
S277 Type VII RTKs: Neurotrophin receptor/Trk family
S278 Type VIII RTKs: ROR family
S278 Type IX RTKs: MuSK
S279 Type X RTKs: HGF (hepatocyte growth factor) receptor family
S279 Type XI RTKs: TAM (TYRO3‐, AXL‐ and MER‐TK) receptor family
S280 Type XII RTKs: TIE family of angiopoietin receptors
S280 Type XIII RTKs: Ephrin receptor family
S281 Type XIV RTKs: RET
S282 Type XV RTKs: RYK
S282 Type XVI RTKs: DDR (collagen receptor) family
S283 Type XVII RTKs: ROS receptors
S283 Type XVIII RTKs: LMR family
S284 Type XIX RTKs: Leukocyte tyrosine kinase (LTK) receptor family
S284 Type XX RTKs: STYK1
S286 Receptor serine/threonine kinase (RSTK) family
S286 Type I receptor serine/threonine kinases
S287 Type II receptor serine/threonine kinases
S287 Type III receptor serine/threonine kinases
S287 RSTK functional heteromers
S289 Receptor tyrosine phosphatase (RTP) family
S291 Tumour necrosis factor (TNF) receptor family

#### S297 Enzymes

S301 Acetylcholine turnover
S302 Adenosine turnover
S303 Amino acid hydroxylases
S304 L‐Arginine turnover
S304 2.1.1.‐ Protein arginine N‐methyltransferases
S305 Arginase
S305 Arginine:glycine amidinotransferase
S305 Dimethylarginine dimethylaminohydrolases
S306 Nitric oxide synthases
S307 Carbonic anhydrases
S308 Carboxylases and decarboxylases
S308 Carboxylases
S309 Decarboxylases
S311 Catecholamine turnover
S313 Ceramide turnover
S313 Serine palmitoyltransferase
S314 Ceramide synthase
S314 Sphingolipid Δ^4^‐desaturase
S315 Sphingomyelin synthase
S315 Sphingomyelin phosphodiesterase
S316 Neutral sphingomyelinase coupling factors
S316 Ceramide glucosyltransferase
S316 Acid ceramidase
S317 Neutral ceramidases
S317 Alkaline ceramidases
S318 Ceramide kinase
S319 Chromatin modifying enzymes
S319 2.1.1.‐ Protein arginine N‐methyltransferases
S320 3.5.1.‐ Histone deacetylases (HDACs)
S321 Cyclic nucleotide turnover/signalling
S321 Adenylyl cyclases (ACs)
S323 Exchange protein activated by cyclic AMP (EPACs)
S323 Phosphodiesterases, 3’,5’‐cyclic nucleotide (PDEs)
S327 Cytochrome P450
S327 CYP2 family
S328 CYP2 family
S329 CYP3 family
S330 CYP4 family
S331 CYP5, CYP7 and CYP8 families
S332 CYP11, CYP17, CYP19, CYP20 and CYP21 families
S333 CYP24, CYP26 and CYP27 families
S333 CYP39, CYP46 and CYP51 families
S334 DNA topoisomerases
S335 Endocannabinoid turnover
S336 *N*‐Acylethanolamine turnover
S337 2‐Acylglycerol ester turnover
S338 Eicosanoid turnover
S338 Cyclooxygenase
S339 Prostaglandin synthases
S341 Lipoxygenases
S342 Leukotriene and lipoxin metabolism
S343 GABA turnover
S344 Glycerophospholipid turnover
S345 Phosphoinositide‐specific phospholipase C
S346 Phospholipase A_2_
S348 Phosphatidylcholine‐specific phospholipase D
S349 Lipid phosphate phosphatases
S349 Phosphatidylinositol kinases
S350 1‐phosphatidylinositol 4‐kinase family
S351 Phosphatidylinositol‐4‐phosphate 3‐kinase family
S351 Phosphatidylinositol 3‐kinase family
S351 Phosphatidylinositol‐4,5‐bisphosphate 3‐kinase family
S352 1‐phosphatidylinositol‐3‐phosphate 5‐kinase family
S353 Type I PIP kinases (1‐phosphatidylinositol‐4‐phosphate 5‐kinase family)
S353 Type II PIP kinases (1‐phosphatidylinositol‐5‐phosphate 4‐kinase family)
S356 Phosphatidylinositol phosphate kinases
S356 Haem oxygenase
S358 Hydrogen sulphide synthesis
S358 Hydrolases
S360 Inositol phosphate turnover
S360 Inositol 1,4,5‐trisphosphate 3‐kinases
S360 Inositol polyphosphate phosphatases
S361 Inositol monophosphatase
S361 Kinases (EC 2.7.x.x)
S362 Rho kinase
S362 Protein kinase C (PKC) family
S363 Alpha subfamily
S363 Delta subfamily
S364 Eta subfamily
S364 FRAP subfamily
S365 Cyclin‐dependent kinase (CDK) family
S365 CDK4 subfamily
S366 GSK subfamily
S367 Polo‐like kinase (PLK) family
S367 STE7 family
S368 Abl family
S368 Ack family
S369 Janus kinase (JakA) family
S369 Src family
S370 Tec family
S371 RAF family
S372 Lanosterol biosynthesis pathway
S374 Nucleoside synthesis and metabolism
S376 Paraoxonase (PON) family
S377 Peptidases and proteinases
S377 A1: Pepsin
S377 A22: Presenilin
S378 C14: Caspase
S378 M1: Aminopeptidase N
S379 M2: Angiotensin‐converting (ACE and ACE2)
S379 M10: Matrix metallopeptidase
S380 M12: Astacin/Adamalysin
S380 M28: Aminopeptidase Y
S381 M19: Membrane dipeptidase
S381 S1: Chymotrypsin
S382 T1: Proteasome
S382 S8: Subtilisin
S383 S9: Prolyl oligopeptidase
S383 Poly ADP‐ribose polymerases
S384 Prolyl hydroxylases
S384 Sphingosine 1‐phosphate turnover
S385 Sphingosine kinase
S386 Sphingosine 1‐phosphate phosphatase
S387 Sphingosine 1‐phosphate lyase
S387 Thyroid hormone turnover
S388 1.14.13.9 Kynurenine 3‐monooxygenase
S389 2.5.1.58 Protein farnesyltransferase
S390 3.5.1.‐ Histone deacetylases (HDACs)
S391 3.5.3.15 Peptidyl arginine deiminases (PADI)
S391 3.6.5.2 Small monomeric GTPases
S391 RAS subfamily
S392 RAB subfamily

#### S397 Transporters

S399 ATP‐binding cassette transporter family
S399 ABCA subfamily
S401 ABCB subfamily
S403 ABCC subfamily
S404 ABCD subfamily of peroxisomal ABC transporters
S405 ABCG subfamily
S406 F‐type and V‐type ATPases
S406 F‐type ATPase
S407 V‐type ATPase
S407 P‐type ATPases
S407 Na^+^/K^+^‐ATPases
S408 Ca^2+^‐ATPases
S408 H^+^/K^+^‐ATPases
S408 Cu^+^‐ATPases
S409 Phospholipid‐transporting ATPases
S409 SLC superfamily of solute carriers
S410 SLC1 family of amino acid transporters
S410 Glutamate transporter subfamily
S412 Alanine/serine/cysteine transporter subfamily
S413 SLC2 family of hexose and sugar alcohol transporters
S413 Class I transporters
S414 Class II transporters
S415 Proton‐coupled inositol transporter
S415 SLC3 and SLC7 families of heteromeric amino acid transporters (HATs)
S415 SLC3 family
S416 SLC7 family
S417 SLC4 family of bicarbonate transporters
S417 Anion exchangers
S418 Sodium‐dependent HCO_3_^−^ transporters
S418 SLC5 family of sodium‐dependent glucose transporters
S419 Hexose transporter family
S420 Choline transporter
S421 Sodium iodide symporter, sodium‐dependent multivitamin transporter and sodium‐coupled monocarboxylate transporters
S422 Sodium *myo*‐inositol cotransporter transporters
S423 SLC6 neurotransmitter transporter family
S423 Monoamine transporter subfamily
S424 GABA transporter subfamily
S425 Glycine transporter subfamily
S427 Neutral amino acid transporter subfamily
S428 SLC8 family of sodium/calcium exchangers
S429 SLC9 family of sodium/hydrogen exchangers
S429 SLC10 family of sodium‐bile acid co‐transporters
S431 SLC11 family of proton‐coupled metal ion transporters
S431 SLC12 family of cation‐coupled chloride transporters
S433 SLC13 family of sodium‐dependent sulphate/carboxylate transporters
S434 SLC14 family of facilitative urea transporters
S435 SLC15 family of peptide transporters
S437 SLC16 family of monocarboxylate transporters
S438 SLC17 phosphate and organic anion transporter family
S438 Type I sodium‐phosphate co‐transporters
S439 Sialic acid transporter
S439 Vesicular glutamate transporters (VGLUTs)
S440 Vesicular nucleotide transporter
S440 SLC18 family of vesicular amine transporters
S442 SLC19 family of vitamin transporters
S443 SLC20 family of sodium‐dependent phosphate transporters
S443 SLC22 family of organic cation and anion transporters
S444 Organic cation transporters (OCT)
S445 Organic zwitterions/cation transporters (OCTN)
S446 Organic anion transporters (OATs)
S446 Urate transporter
S447 Atypical SLC22B subfamily
S448 SLC23 family of ascorbic acid transporters
S449 SLC24 family of sodium/potassium/calcium exchangers
S450 SLC25 family of mitochondrial transporters
S450 Mitochondrial di‐ and tri‐carboxylic acid transporter subfamily
S451 Mitochondrial amino acid transporter subfamily
S452 Mitochondrial phosphate transporters
S452 Mitochondrial nucleotide transporter subfamily
S453 Mitochondrial uncoupling proteins
S454 Miscellaneous SLC25 mitochondrial transporters
S454 SLC26 family of anion exchangers
S454 Selective sulphate transporters
S455 Chloride/bicarbonate exchangers
S455 Anion channels
S456 Other SLC26 anion exchangers
S457 SLC27 family of fatty acid transporters
S458 SLC28 and SLC29 families of nucleoside transporters
S458 SLC28 family
S459 SLC29 family
S461 SLC30 zinc transporter family
S461 SLC31 family of copper transporters
S462 SLC32 vesicular inhibitory amino acid transporter
S463 SLC33 acetylCoA transporter
S464 SLC34 family of sodium phosphate co‐transporters
S465 SLC35 family of nucleotide sugar transporters
S466 SLC36 family of proton‐coupled amino acid transporters
S468 SLC37 family of phosphosugar/phosphate exchangers
S468 SLC38 family of sodium‐dependent neutral amino acid transporters
S469 System A‐like transporters
S469 System N‐like transporters
S470 Orphan SLC38 transporters
S470 SLC39 family of metal ion transporters
S471 SLC40 iron transporter
S472 SLC41 family of divalent cation transporters
S473 SLC42 family of Rhesus glycoprotein ammoniumtransporters
S473 SLC43 family of large neutral amino acid transporters
S474 SLC44 choline transporter‐like family
S475 SLC45 family of putative sugar transporters
S475 SLC46 family of folate transporters
S477 SLC47 family of multidrug and toxin extrusion transporters
S477 SLC48 heme transporter
S478 SLC49 family of FLVCR‐related heme transporters
S479 SLC50 sugar transporter
S479 SLC51 family of steroid‐derived molecule transporters
S480 SLC52 family of riboflavin transporters
S481 SLC53 Phosphate carriers
S481 SLC54 Mitochondrial pyruvate carriers
S482 SLC55 Mitochondrial cation/proton exchangers
S482 SLC56 Sideroflexins
S483 SLC57 NiPA‐like magnesium transporter family
S483 SLC58 MagT‐like magnesium transporter family
S484 SLC59 Sodium‐dependent lysophosphatidylcholine symporter family
S484 SLC60 Glucose transporters
S485 SLC61 Molybdate transporter family
S485 SLC62 Pyrophosphate transporters
S486 SLC63 Sphingosine phosphate transporters
S486 SLC64 Golgi Ca2+/H+ exchangers
S487 SLC65 NPC‐type cholesterol transporters
S488 SLCO family of organic anion transporting polypeptides

### Introduction

In order to allow clarity and consistency in pharmacology, there is a need for a comprehensive organisation and presentation of the targets of drugs. This is the philosophy of the IUPHAR/BPS Guide to PHARMACOLOGY presented on the online free access database (https://www.guidetopharmacology.org/). This database is supported by the British Pharmacological Society (BPS), the International Union of Basic and Clinical Pharmacology (IUPHAR), the University of Edinburgh and previously the Wellcome Trust. Data included in the Guide to PHARMACOLOGY are derived in large part from interactions with the subcommittees of the Nomenclature Committee of the International Union of Basic and Clinical Pharmacology (NC‐IUPHAR). A major influence on the development of the database was Tony Harmar (1951‐2014), who worked with a passion to establish the curators as a team of highly informed and informative individuals, with a focus on high‐quality data input, ensuring a suitably validated dataset. The Editors of the Concise Guide have compiled the individual records, in concert with the team of Curators, drawing on the expert knowledge of these latter subcommittees. The tables allow an indication of the status of the nomenclature for the group of targets listed, usually previously published in Pharmacological Reviews. In the absence of an established subcommittee, advice from several prominent, independent experts has generally been obtained to produce an authoritative consensus on nomenclature, which attempts to fit in within the general guidelines from NC‐IUPHAR. This current edition, the Concise Guide to PHARMACOLOGY 2019/20, is the latest snapshot of the database in print form, following on from the Concise Guide to PHARMACOLOGY 2017/18. It contains data drawn from the online database as a rapid overview of the major pharmacological targets. Thus, there are many fewer targets presented in the Concise Guide compared to the online database. The priority for inclusion in the Concise Guide is the presence of quantitative pharmacological data for human proteins. This means that often orphan family members are not presented in the Concise Guide, although structural information is available on the online database. The organisation of the data is tabular (where appropriate) with a standardised format, where possible on a single page, intended to aid understanding of, and comparison within, a particular target group. The Concise Guide is intended as an initial resource, with links to additional reviews and resources for greater depth and information. Pharmacological and structural data focus primarily on human gene products, wherever possible, with links to HGNC gene nomenclature and UniProt IDs. In a few cases, where data from human proteins are limited, data from other species are indicated. Pharmacological tools listed are prioritised on the basis of selectivity and availability. That is, agents (agonists, antagonists, inhibitors, activators, etc.) are included where they are both available (by donation or from commercial sources, now or in the near future) AND the most selective. The Concise Guide is divided into seven sections, which comprise pharmacological targets of similar structure/function. These are G protein‐coupled receptors, ion channels (combining previous records of ligand‐gated, voltage‐gated and other ion channels), catalytic receptors, nuclear hormone receptors, enzymes, transporters and other protein targets. We hope that the Concise Guide will provide for researchers, teachers and students a state‐of‐the art source of accurate, curated information on the background to their work that they will use in the Introductions to their Research Papers or Reviews, or in supporting their teaching and studies. We recommend that any citations to information in the Concise Guide are presented in the following format: Alexander SPH et al. (2019). The Concise Guide to PHARMACOLOGY 2019/20: Introduction and Other Protein Targets. Br J Pharmacol 176: S1–S20. In this overview are listed protein targets of pharmacological interest, which are not G protein‐coupled receptors, ion channels, nuclear hormone receptors, catalytic receptors, transporters or enzymes.

### Acknowledgements

We are extremely grateful to the British Pharmacological Society and the International Union of Basic and Clinical Pharmacology, for financial support of the website and for advice from the NC‐IUPHAR subcommittees. We thank the University of Edinburgh, who host the http://www.guidetopharmacology.org website. Previously, the International Union of Basic and Clinical Pharmacology and the Wellcome Trust (099156/Z/12/Z]) also supported the initiation and expansion of the database. We are also tremendously grateful to the long list of collaborators from NC‐IUPHAR subcommittees and beyond, who have assisted in the construction of the Concise Guide to PHARMACOLOGY 2019/20 and the online database http://www.guidetopharmacology.org.

### Conflict of interest

The authors state that there are no conflicts of interest to disclose.

### Family structure

– http://www.guidetopharmacology.org/GRAC/FamilyDisplayForward?familyId=1021

S6 Adiponectin receptors

– http://www.guidetopharmacology.org/GRAC/FamilyDisplayForward?familyId=982

– http://www.guidetopharmacology.org/GRAC/FamilyDisplayForward?familyId=970

– http://www.guidetopharmacology.org/GRAC/FamilyDisplayForward?familyId=983

– http://www.guidetopharmacology.org/GRAC/FamilyDisplayForward?familyId=971

– http://www.guidetopharmacology.org/GRAC/FamilyDisplayForward?familyId=910

S7 Blood coagulation components

– http://www.guidetopharmacology.org/GRAC/FamilyDisplayForward?familyId=866

S8 Non‐enzymatic BRD containing proteins

– http://www.guidetopharmacology.org/GRAC/FamilyDisplayForward?familyId=966

S8 Carrier proteins

S9 CD molecules

– http://www.guidetopharmacology.org/GRAC/FamilyDisplayForward?familyId=997

– http://www.guidetopharmacology.org/GRAC/FamilyDisplayForward?familyId=998

– http://www.guidetopharmacology.org/GRAC/FamilyDisplayForward?familyId=986

– http://www.guidetopharmacology.org/GRAC/FamilyDisplayForward?familyId=901

S11 Methyllysine reader proteins

– http://www.guidetopharmacology.org/GRAC/FamilyDisplayForward?familyId=916

– http://www.guidetopharmacology.org/GRAC/FamilyDisplayForward?familyId=930

– http://www.guidetopharmacology.org/GRAC/FamilyDisplayForward?familyId=915

S11 Fatty acid-binding proteins

– http://www.guidetopharmacology.org/GRAC/FamilyDisplayForward?familyId=946

– http://www.guidetopharmacology.org/GRAC/FamilyDisplayForward?familyId=935

– http://www.guidetopharmacology.org/GRAC/FamilyDisplayForward?familyId=784

– http://www.guidetopharmacology.org/GRAC/FamilyDisplayForward?familyId=954

– http://www.guidetopharmacology.org/GRAC/FamilyDisplayForward?familyId=949

– http://www.guidetopharmacology.org/GRAC/FamilyDisplayForward?familyId=985

– http://www.guidetopharmacology.org/GRAC/FamilyDisplayForward?familyId=987

– http://www.guidetopharmacology.org/GRAC/FamilyDisplayForward?familyId=984

– http://www.guidetopharmacology.org/GRAC/FamilyDisplayForward?familyId=868

– http://www.guidetopharmacology.org/GRAC/FamilyDisplayForward?familyId=889

– http://www.guidetopharmacology.org/GRAC/FamilyDisplayForward?familyId=876

– http://www.guidetopharmacology.org/GRAC/FamilyDisplayForward?familyId=888

– http://www.guidetopharmacology.org/GRAC/FamilyDisplayForward?familyId=934

– http://www.guidetopharmacology.org/GRAC/FamilyDisplayForward?familyId=924

– http://www.guidetopharmacology.org/GRAC/FamilyDisplayForward?familyId=919

– http://www.guidetopharmacology.org/GRAC/FamilyDisplayForward?familyId=921

– http://www.guidetopharmacology.org/GRAC/FamilyDisplayForward?familyId=991

– http://www.guidetopharmacology.org/GRAC/FamilyDisplayForward?familyId=941

– http://www.guidetopharmacology.org/GRAC/FamilyDisplayForward?familyId=942

– http://www.guidetopharmacology.org/GRAC/FamilyDisplayForward?familyId=945

– http://www.guidetopharmacology.org/GRAC/FamilyDisplayForward?familyId=929

S14 Notch receptors

– http://www.guidetopharmacology.org/GRAC/FamilyDisplayForward?familyId=1001

– http://www.guidetopharmacology.org/GRAC/FamilyDisplayForward?familyId=906

– http://www.guidetopharmacology.org/GRAC/FamilyDisplayForward?familyId=907

S15 Regulators of G protein Signaling (RGS) proteins

S15 RZ family

S15 R4 family

S16 R7 family

S17 R12 family

– http://www.guidetopharmacology.org/GRAC/FamilyDisplayForward?familyId=932

– http://www.guidetopharmacology.org/GRAC/FamilyDisplayForward?familyId=905

– http://www.guidetopharmacology.org/GRAC/FamilyDisplayForward?familyId=875

– http://www.guidetopharmacology.org/GRAC/FamilyDisplayForward?familyId=1000

S18 Sigma receptors

– http://www.guidetopharmacology.org/GRAC/FamilyDisplayForward?familyId=989

– http://www.guidetopharmacology.org/GRAC/FamilyDisplayForward?familyId=972

– http://www.guidetopharmacology.org/GRAC/FamilyDisplayForward?familyId=1019

– http://www.guidetopharmacology.org/GRAC/FamilyDisplayForward?familyId=973

– http://www.guidetopharmacology.org/GRAC/FamilyDisplayForward?familyId=974

– http://www.guidetopharmacology.org/GRAC/FamilyDisplayForward?familyId=990

– http://www.guidetopharmacology.org/GRAC/FamilyDisplayForward?familyId=995

– http://www.guidetopharmacology.org/GRAC/FamilyDisplayForward?familyId=996

S19 Tubulins

– http://www.guidetopharmacology.org/GRAC/FamilyDisplayForward?familyId=904

– http://www.guidetopharmacology.org/GRAC/FamilyDisplayForward?familyId=903

### http://www.guidetopharmacology.org/GRAC/FamilyDisplayForward?familyId=106

#### Overview

Adiponectin receptors (**provisional nomenclature**, http://www.ensembl.org/Homo_sapiens/Gene/Family/Genes?family=ENSFM00500000270960) respond to the 30 kDa complement‐related protein hormone adiponectin (also known as https://www.genenames.org/data/gene-symbol-report/#!/hgnc_id/HGNC:13633: adipocyte, C1q and collagen domain‐containing protein; ACRP30, adipose most abundant gene transcript 1; apM‐1; gelatin‐binding protein: http://www.uniprot.org/uniprot/Q15848) originally cloned from adipocytes [http://www.ncbi.nlm.nih.gov/pubmed/8619847?dopt=AbstractPlus]. Although sequence data suggest 7TM domains, immunological evidence indicates that, contrary to typical 7TM topology, the carboxyl terminus is extracellular, while the amino terminus is intracellular [http://www.ncbi.nlm.nih.gov/pubmed/12802337?dopt=AbstractPlus]. Signalling through these receptors appears to avoid G proteins; modelling based on the crystal structures of the adiponectin receptors suggested ceramidase acivity, which would make these the first in a new family of catalytic receptors [http://www.ncbi.nlm.nih.gov/pubmed/25855295?dopt=AbstractPlus].

**Table:** 

Nomenclature	http://www.guidetopharmacology.org/GRAC/ObjectDisplayForward?objectId=649	http://www.guidetopharmacology.org/GRAC/ObjectDisplayForward?objectId=650
HGNC, UniProt	https://www.genenames.org/data/gene-symbol-report/#!/hgnc_id/HGNC:24040, http://www.uniprot.org/uniprot/Q96A54	https://www.genenames.org/data/gene-symbol-report/#!/hgnc_id/HGNC:24041, http://www.uniprot.org/uniprot/Q86V24
Rank order of potency	http://www.guidetopharmacology.org/GRAC/LigandDisplayForward?ligandId=3727 (https://www.genenames.org/data/gene-symbol-report/#!/hgnc_id/HGNC:13633, http://www.uniprot.org/uniprot/Q15848) > http://www.guidetopharmacology.org/GRAC/LigandDisplayForward?ligandId=3726 (https://www.genenames.org/data/gene-symbol-report/#!/hgnc_id/HGNC:13633, http://www.uniprot.org/uniprot/Q15848)	http://www.guidetopharmacology.org/GRAC/LigandDisplayForward?ligandId=3727 (https://www.genenames.org/data/gene-symbol-report/#!/hgnc_id/HGNC:13633, http://www.uniprot.org/uniprot/Q15848) = http://www.guidetopharmacology.org/GRAC/LigandDisplayForward?ligandId=3726 (https://www.genenames.org/data/gene-symbol-report/#!/hgnc_id/HGNC:13633, http://www.uniprot.org/uniprot/Q15848)

#### Comments

T‐Cadherin (https://www.genenames.org/data/gene-symbol-report/#!/hgnc_id/HGNC:1753, http://www.uniprot.org/uniprot/P55290) has also been suggested to be a receptor for (hexameric) adiponectin [http://www.ncbi.nlm.nih.gov/pubmed/15210937?dopt=AbstractPlus].

#### Further reading on Adiponectin receptors

Fisman EZ *et al*. (2014) Adiponectin: a manifold therapeutic target for metabolic syndrome, diabetes, and coronary disease? *Cardiovasc Diabetol* **13**: 103 [https://www.ncbi.nlm.nih.gov/pubmed/24957699?dopt=AbstractPlus]

Okada‐Iwabu M *et al*. (2018) Structure and function analysis of adiponectin receptors toward development of novel antidiabetic agents promoting healthy longevity. *Endocr J* **65**: 971‐977 [https://www.ncbi.nlm.nih.gov/pubmed/30282888]

Ruan H *et al*. (2016) Adiponectin signaling and function in insulin target tissues. *J Mol Cell Biol* **8**: 101‐9 [https://www.ncbi.nlm.nih.gov/pubmed/26993044?dopt=AbstractPlus]

Wang Y *et al*. (2017) Cardiovascular Adiponectin Resistance: The Critical Role of Adiponectin Receptor Modification. *Trends Endocrinol. Metab.* **28**: 519‐530 [https://www.ncbi.nlm.nih.gov/pubmed/28473178?dopt=AbstractPlus]

Zhao L *et al*. (2014) Adiponectin and insulin cross talk: the microvascular connection. *Trends Cardiovasc. Med.* **24**: 319‐24 [https://www.ncbi.nlm.nih.gov/pubmed/25220977?dopt=AbstractPlus]

### http://www.guidetopharmacology.org/GRAC/FamilyDisplayForward?familyId=853

#### Overview

Coagulation as a process is interpreted as a mechanism for reducing excessive blood loss through the generation of a gel‐like clot local to the site of injury. The process involves the activation, adhesion (see http://www.guidetopharmacology.org/GRAC/FamilyDisplayForward?familyId=760), degranulation and aggregation of platelets, as well as proteins circulating in the plasma. The coagulation cascade involves multiple proteins being converted to more active forms from less active precursors, typically through proteolysis (see http://www.guidetopharmacology.org/GRAC/FamilyDisplayForward?familyId=759&familyType=ENZYME). Listed here are the components of the coagulation cascade targetted by agents in current clinical usage.

**Table:** 

Nomenclature	http://www.guidetopharmacology.org/GRAC/ObjectDisplayForward?objectId=2606	http://www.guidetopharmacology.org/GRAC/ObjectDisplayForward?objectId=2607	http://www.guidetopharmacology.org/GRAC/ObjectDisplayForward?objectId=2632
HGNC, UniProt	https://www.genenames.org/data/gene-symbol-report/#!/hgnc_id/HGNC:3542, http://www.uniprot.org/uniprot/P12259	https://www.genenames.org/data/gene-symbol-report/#!/hgnc_id/HGNC:3546, http://www.uniprot.org/uniprot/P00451	https://www.genenames.org/data/gene-symbol-report/#!/hgnc_id/HGNC:775, http://www.uniprot.org/uniprot/P01008
Selective activators	–	–	http://www.guidetopharmacology.org/GRAC/LigandDisplayForward?ligandId=4214 (p*K*_d_ 7.8) [http://www.ncbi.nlm.nih.gov/pubmed/23598032?dopt=AbstractPlus], http://www.guidetopharmacology.org/GRAC/LigandDisplayForward?ligandId=6819 (p*K*_d_ 7.5) [http://www.ncbi.nlm.nih.gov/pubmed/12383040?dopt=AbstractPlus], http://www.guidetopharmacology.org/GRAC/LigandDisplayForward?ligandId=6803 [http://www.ncbi.nlm.nih.gov/pubmed/3744129?dopt=AbstractPlus], http://www.guidetopharmacology.org/GRAC/LigandDisplayForward?ligandId=6804 [http://www.ncbi.nlm.nih.gov/pubmed/8137606?dopt=AbstractPlus, http://www.ncbi.nlm.nih.gov/pubmed/19398784?dopt=AbstractPlus], http://www.guidetopharmacology.org/GRAC/LigandDisplayForward?ligandId=6811 [http://www.ncbi.nlm.nih.gov/pubmed/7667822?dopt=AbstractPlus], http://www.guidetopharmacology.org/GRAC/LigandDisplayForward?ligandId=6846 [http://www.ncbi.nlm.nih.gov/pubmed/7528134?dopt=AbstractPlus]
Selective inhibitors	http://www.guidetopharmacology.org/GRAC/LigandDisplayForward?ligandId=6788 (Antithrombotic effect thought to occur via inhibition of factors Va and VIIIa) [http://www.ncbi.nlm.nih.gov/pubmed/11714212?dopt=AbstractPlus, http://www.ncbi.nlm.nih.gov/pubmed/11463021?dopt=AbstractPlus]	http://www.guidetopharmacology.org/GRAC/LigandDisplayForward?ligandId=6788 (Antithrombotic effect thought to occur via inhibition of factors Va and VIIIa) [http://www.ncbi.nlm.nih.gov/pubmed/11714212?dopt=AbstractPlus, http://www.ncbi.nlm.nih.gov/pubmed/11463021?dopt=AbstractPlus]	–

#### Further reading on Blood coagulation components

Astermark J. (2015) FVIII inhibitors: pathogenesis and avoidance. *Blood* **125**: 2045‐51 [https://www.ncbi.nlm.nih.gov/pubmed/25712994?dopt=AbstractPlus]

Girolami A *et al*. (2017) New clotting disorders that cast new light on blood coagulation and may play a role in clinical practice. *J. Thromb. Thrombolysis* **44**: 71‐75 [https://www.ncbi.nlm.nih.gov/pubmed/28251495?dopt=AbstractPlus]

Rana K *et al*. (2016) Blood flow and mass transfer regulation of coagulation. *Blood Rev.* **30**: 357‐68 [https://www.ncbi.nlm.nih.gov/pubmed/27133256?dopt=AbstractPlus]

### http://www.guidetopharmacology.org/GRAC/FamilyDisplayForward?familyId=867

#### Overview

Bromodomains bind proteins with acetylated lysine residues, such as histones, to regulate gene transcription. Listed herein are examples of bromodomain‐containing proteins for which sufficient pharmacology exists.

**Table:** 

Nomenclature	http://www.guidetopharmacology.org/GRAC/ObjectDisplayForward?objectId=2721	http://www.guidetopharmacology.org/GRAC/ObjectDisplayForward?objectId=2722	http://www.guidetopharmacology.org/GRAC/ObjectDisplayForward?objectId=2734	http://www.guidetopharmacology.org/GRAC/ObjectDisplayForward?objectId=2738	http://www.guidetopharmacology.org/GRAC/ObjectDisplayForward?objectId=2740
HGNC, UniProt	https://www.genenames.org/data/gene-symbol-report/#!/hgnc_id/HGNC:962, http://www.uniprot.org/uniprot/Q9UIF9	https://www.genenames.org/data/gene-symbol-report/#!/hgnc_id/HGNC:963, http://www.uniprot.org/uniprot/Q9UIF8	https://www.genenames.org/data/gene-symbol-report/#!/hgnc_id/HGNC:2348, http://www.uniprot.org/uniprot/Q92793	https://www.genenames.org/data/gene-symbol-report/#!/hgnc_id/HGNC:30064, http://www.uniprot.org/uniprot/Q86U86	https://www.genenames.org/data/gene-symbol-report/#!/hgnc_id/HGNC:11100, http://www.uniprot.org/uniprot/P51532
Selective inhibitors	http://www.guidetopharmacology.org/GRAC/LigandDisplayForward?ligandId=8233 (p*K*_d_ 6.6) [85]	http://www.guidetopharmacology.org/GRAC/LigandDisplayForward?ligandId=8233 (Binding) (p*K*_d_ 6.9) [85]	http://www.guidetopharmacology.org/GRAC/LigandDisplayForward?ligandId=8236 (p*K*_d_ 6.8) [84]	http://www.guidetopharmacology.org/GRAC/LigandDisplayForward?ligandId=7915 (Binding) (p*K*_d_ 7.3) [95]	http://www.guidetopharmacology.org/GRAC/LigandDisplayForward?ligandId=7915 (Binding) (p*K*_d_ 7.1) [95]

#### Further reading on Non‐enzymatic BRD containing proteins

Fujisawa T *et al*. (2017) Functions of bromodomain‐containing proteins and their roles in homeostasis and cancer. *Nat. Rev. Mol. Cell Biol.* **18**: 246‐262 [https://www.ncbi.nlm.nih.gov/pubmed/28053347?dopt=AbstractPlus]

Myrianthopoulos V & Mikros E. (2019) From bench to bedside, via desktop. Recent advances in the application of cutting‐edge in silico tools in the research of drugs targeting bromodomain modules. *Biochem Pharmacol* **159**: 40‐51 [https://www.ncbi.nlm.nih.gov/pubmed/30414936]

Nicholas DA *et al*. (2017) BET bromodomain proteins and epigenetic regulation of inflammation: implications for type 2 diabetes and breast cancer. *Cell. Mol. Life Sci.* **74**: 231‐243 [https://www.ncbi.nlm.nih.gov/pubmed/27491296?dopt=AbstractPlus]

Ramadoss M & Mahadevan V. (2018) Targeting the cancer epigenome: synergistic therapy with bromodomain inhibitors. *Drug Discov Today* **23**: 76‐89 [https://www.ncbi.nlm.nih.gov/pubmed/28943305]

Yang CY *et al*. (2019) Small‐molecule PROTAC degraders of the Bromodomain and Extra Terminal (BET) proteins ‐ A review. *Drug Discov Today Technol* **31**: 43‐51 [https://www.ncbi.nlm.nih.gov/pubmed/31200858]

### http://www.guidetopharmacology.org/GRAC/FamilyDisplayForward?familyId=911

#### Overview

Transthyretin (TTR) is a homo‐tetrameric protein which transports thyroxine in the plasma and cerebrospinal fluid and retinol (vitamin A) in the plasma. Many disease causing mutations in the protein have been reported, many of which cause complex dissociation and protein mis‐assembly and deposition of toxic aggregates amyloid fibril formation [http://www.ncbi.nlm.nih.gov/pubmed/23716704?dopt=AbstractPlus]. These amyloidogenic mutants are linked to the development of pathological amyloidoses, including familial amyloid polyneuropathy (FAP) [http://www.ncbi.nlm.nih.gov/pubmed/12978172?dopt=AbstractPlus, http://www.ncbi.nlm.nih.gov/pubmed/8894411?dopt=AbstractPlus], familial amyloid cardiomyopathy (FAC) [http://www.ncbi.nlm.nih.gov/pubmed/9017939?dopt=AbstractPlus], amyloidotic vitreous opacities, carpal tunnel syndrome [http://www.ncbi.nlm.nih.gov/pubmed/10403814?dopt=AbstractPlus] and others. In old age, non‐mutated TTR can also form pathological amyloid fibrils [http://www.ncbi.nlm.nih.gov/pubmed/7016817?dopt=AbstractPlus]. Pharmacological intervention to reduce or prevent TTR dissociation is being pursued as a theapeutic strategy. To date one small molecule kinetic stabilising molecule (http://www.guidetopharmacology.org/GRAC/LigandDisplayForward?ligandId=8378) has been approved for FAP, and is being evaluated in clinical trials for other TTR amyloidoses.

**Table:** 

Nomenclature	http://www.guidetopharmacology.org/GRAC/ObjectDisplayForward?objectId=2851
Common abbreviation	TTR
HGNC, UniProt	https://www.genenames.org/data/gene-symbol-report/#!/hgnc_id/HGNC:12405, http://www.uniprot.org/uniprot/P02766

#### Further reading on Carrier proteins

Adams D *et al*. (2019) Hereditary transthyretin amyloidosis: a model of medical progress for a fatal disease. *Nat Rev Neurol* **15**: 387‐404 [https://www.ncbi.nlm.nih.gov/pubmed/31209302]

Dellière S *et al*. (2017) Is transthyretin a good marker of nutritional status? *Clin Nutr* **36**: 364‐370 [https://www.ncbi.nlm.nih.gov/pubmed/27381508?dopt=AbstractPlus]

Galant NJ *et al*. (2017) Transthyretin amyloidosis: an under‐recognized neuropathy and cardiomyopathy. *Clin. Sci.* **131**: 395‐409 [https://www.ncbi.nlm.nih.gov/pubmed/28213611?dopt=AbstractPlus]

Yokoyama T & Mizuguchi M. (2018) Inhibition of the Amyloidogenesis of Transthyretin by Natural Products and Synthetic Compounds. *Biol Pharm Bull* **41**: 979‐984 [https://www.ncbi.nlm.nih.gov/pubmed/29962408]

Ruberg FL *et al*. (2019) Transthyretin Amyloid Cardiomyopathy: JACC State‐of‐the‐Art Review. *J Am Coll Cardiol* **73**: 2872‐2891 [https://www.ncbi.nlm.nih.gov/pubmed/31171094]

### http://www.guidetopharmacology.org/GRAC/FamilyDisplayForward?familyId=852

#### Overview

Cluster of differentiation refers to an attempt to catalogue systematically a series of over 300 cell‐surface proteins associated with immunotyping. Many members of the group have identified functions as enzymes (for example, see http://www.guidetopharmacology.org/GRAC/ObjectDisplayForward?objectId=1232) or receptors (for example, see http://www.guidetopharmacology.org/GRAC/ObjectDisplayForward?objectId=2441). Many CDs are targeted for therapeutic gain using antibodies for the treatment of proliferative disorders. A full listing of all the Clusters of Differentiation proteins is not possible in the Guide to PHARMACOLOGY; listed herein are selected members of the family targeted for therapeutic gain.

**Table:** 

Nomenclature	http://www.guidetopharmacology.org/GRAC/ObjectDisplayForward?objectId=2600	http://www.guidetopharmacology.org/GRAC/ObjectDisplayForward?objectId=2742	http://www.guidetopharmacology.org/GRAC/ObjectDisplayForward?objectId=2917	http://www.guidetopharmacology.org/GRAC/ObjectDisplayForward?objectId=2628)	http://www.guidetopharmacology.org/GRAC/ObjectDisplayForward?objectId=2601
Common abbreviation	–	–	–	–	SIGLEC‐3
HGNC, UniProt	https://www.genenames.org/data/gene-symbol-report/#!/hgnc_id/HGNC:1639, http://www.uniprot.org/uniprot/P06729	https://www.genenames.org/data/gene-symbol-report/#!/hgnc_id/HGNC:1674, http://www.uniprot.org/uniprot/P07766	https://www.genenames.org/data/gene-symbol-report/#!/hgnc_id/HGNC:1691, http://www.uniprot.org/uniprot/P30203	https://www.genenames.org/data/gene-symbol-report/#!/hgnc_id/HGNC:7315, http://www.uniprot.org/uniprot/P11836	https://www.genenames.org/data/gene-symbol-report/#!/hgnc_id/HGNC:1659, http://www.uniprot.org/uniprot/P20138
Selective inhibitors	http://www.guidetopharmacology.org/GRAC/LigandDisplayForward?ligandId=6787 [http://www.ncbi.nlm.nih.gov/pubmed/11970990?dopt=AbstractPlus, http://www.ncbi.nlm.nih.gov/pubmed/12089534?dopt=AbstractPlus]	–	–	–	–
Antibodies	–	http://www.guidetopharmacology.org/GRAC/LigandDisplayForward?ligandId=7385 (Binding) [http://www.ncbi.nlm.nih.gov/pubmed/20190561?dopt=AbstractPlus], http://www.guidetopharmacology.org/GRAC/LigandDisplayForward?ligandId=6889 (Binding) [http://www.ncbi.nlm.nih.gov/pubmed/3105134?dopt=AbstractPlus], http://www.guidetopharmacology.org/GRAC/LigandDisplayForward?ligandId=8458 (Binding) [http://www.ncbi.nlm.nih.gov/pubmed/8436176?dopt=AbstractPlus]	–	http://www.guidetopharmacology.org/GRAC/LigandDisplayForward?ligandId=6778 (Binding) (p*K*_d_ 9.9) [52], http://www.guidetopharmacology.org/GRAC/LigandDisplayForward?ligandId=6780 (Binding) (p*K*_d_ 8.5) [http://www.ncbi.nlm.nih.gov/pubmed/15102696?dopt=AbstractPlus], http://www.guidetopharmacology.org/GRAC/LigandDisplayForward?ligandId=6777 (Binding), http://www.guidetopharmacology.org/GRAC/LigandDisplayForward?ligandId=6941 (Binding) [http://www.ncbi.nlm.nih.gov/pubmed/21378274?dopt=AbstractPlus, http://www.ncbi.nlm.nih.gov/pubmed/23537278?dopt=AbstractPlus], http://www.guidetopharmacology.org/GRAC/LigandDisplayForward?ligandId=6781 (Binding)	http://www.guidetopharmacology.org/GRAC/LigandDisplayForward?ligandId=7983 (Binding) (p*K*_d_ ∼10) [http://www.ncbi.nlm.nih.gov/pubmed/1458463?dopt=AbstractPlus], http://www.guidetopharmacology.org/GRAC/LigandDisplayForward?ligandId=6775 (Binding) [http://www.ncbi.nlm.nih.gov/pubmed/10720144?dopt=AbstractPlus]

**Table:** 

Nomenclature	http://www.guidetopharmacology.org/GRAC/ObjectDisplayForward?objectId=2602	http://www.guidetopharmacology.org/GRAC/ObjectDisplayForward?objectId=2744	http://www.guidetopharmacology.org/GRAC/ObjectDisplayForward?objectId=2745	http://www.guidetopharmacology.org/GRAC/ObjectDisplayForward?objectId=2743)	http://www.guidetopharmacology.org/GRAC/ObjectDisplayForward?objectId=2760)	http://www.guidetopharmacology.org/GRAC/ObjectDisplayForward?objectId=2918
Common abbreviation	–	–	–	CTLA‐4	PD‐1	–
HGNC, UniProt	https://www.genenames.org/data/gene-symbol-report/#!/hgnc_id/HGNC:1804, http://www.uniprot.org/uniprot/P31358	https://www.genenames.org/data/gene-symbol-report/#!/hgnc_id/HGNC:1700, http://www.uniprot.org/uniprot/P33681	https://www.genenames.org/data/gene-symbol-report/#!/hgnc_id/HGNC:1705, http://www.uniprot.org/uniprot/P42081	https://www.genenames.org/data/gene-symbol-report/#!/hgnc_id/HGNC:2505, http://www.uniprot.org/uniprot/P16410	https://www.genenames.org/data/gene-symbol-report/#!/hgnc_id/HGNC:8760, http://www.uniprot.org/uniprot/Q15116	https://www.genenames.org/data/gene-symbol-report/#!/hgnc_id/HGNC:19319, http://www.uniprot.org/uniprot/Q9UGN4
Endogenous ligands	–	–	–	–	http://www.guidetopharmacology.org/GRAC/LigandDisplayForward?ligandId=9606 (https://www.genenames.org/data/gene-symbol-report/#!/hgnc_id/HGNC:17635, https://www.uniprot.org/uniprot/Q9NZQ7) (Binding)	–
Selective inhibitors	–	abatacept (p*K*_d_ ∼7.9) [51, 103]	abatacept (p*K*d ∼7.9) [http://www.guidetopharmacology.org/GRAC/LigandDisplayForward?ligandId=6891], belatacept [44]	–	–	–
Antibodies	alemtuzumab (http://www.guidetopharmacology.org/GRAC/LigandDisplayForward?ligandId=6770, 86]	–	–	ipilimumab (Binding) (http://www.guidetopharmacology.org/GRAC/LigandDisplayForward?ligandId=6888) [30], tremelimumab (Binding) (http://www.guidetopharmacology.org/GRAC/LigandDisplayForward?ligandId=8462]	pembrolizumab (Binding) (p*K*_d_ http://www.guidetopharmacology.org/GRAC/LigandDisplayForward?ligandId=7499 (Binding) (phttp://www.guidetopharmacology.org/GRAC/LigandDisplayForward?ligandId=7499_d_ 9.1) [http://www.guidetopharmacology.org/GRAC/LigandDisplayForward?ligandId=7335]	–

#### Comments

The endogenous ligands for human PD‐1 are programmed cell death 1 ligand 1 (PD‐L1 *aka* http://www.guidetopharmacology.org/GRAC/LigandDisplayForward?ligandId=7693 (https://www.genenames.org/data/gene-symbol-report/#!/hgnc_id/HGNC:17635, http://www.uniprot.org/uniprot/Q9NZQ7)) and programmed cell death 1 ligand 2 (PD‐L2; https://www.genenames.org/data/gene-symbol-report/#!/hgnc_id/HGNC:18731). These ligands are cell surface peptides, normally involved in immune system regulation. Expression of PD‐1 by cancer cells induces immune tolerance and evasion of immune system attack. Anti‐PD‐1 monoclonal antibodies are used to induce immune checkpoint blockade as a therapeutic intervention in cancer, effectively re‐establishing immune vigilance. http://www.guidetopharmacology.org/GRAC/LigandDisplayForward?ligandId=7499 was the first anti‐PD‐1 antibody to be approved by the US FDA.

#### Further reading on CD molecules

Gabius HJ *et al*. (2015) The glycobiology of the CD system: a dictionary for translating marker designations into glycan/lectin structure and function. *Trends Biochem. Sci.* **40**: 360‐76 [https://www.ncbi.nlm.nih.gov/pubmed/25981696?dopt=AbstractPlus]

Vosoughi T *et al*. (2019) CD markers variations in chronic lymphocytic leukemia: New insights into prognosis. *J Cell Physiol*. **234**: 19420‐39 [https://www.ncbi.nlm.nih.gov/pubmed/31049958]

### http://www.guidetopharmacology.org/GRAC/FamilyDisplayForward?familyId=902

#### Overview

Methyllysine reader proteins bind to methylated proteins, such as histones, allowing regulation of gene expression.

**Table:** 

Nomenclature	http://www.guidetopharmacology.org/GRAC/ObjectDisplayForward?objectId=2830
HGNC, UniProt	https://www.genenames.org/data/gene-symbol-report/#!/hgnc_id/HGNC:23035, http://www.uniprot.org/uniprot/Q96JM7
Selective agonists	http://www.guidetopharmacology.org/GRAC/LigandDisplayForward?ligandId=8232 [http://www.ncbi.nlm.nih.gov/pubmed/23292653?dopt=AbstractPlus]

#### Further reading on Methyllysine reader proteins

Daskalaki MG *et al*. (2018) Histone methylation and acetylation in macrophages as a mechanism for regulation of inflammatory responses. *J Cell Physiol*. **233**: 6495‐9507 [https://www.ncbi.nlm.nih.gov/pubmed/29574768]

Furuya K *et al*. (2019) Epigenetic interplays between DNA demethylation and histone methylation for protecting oncogenesis. *J Biochem*. **165**: 297‐299 [https://www.ncbi.nlm.nih.gov/pubmed/30605533]

Levy D. (2019) Lysine methylation signaling of non‐histone proteins in the nucleus. *Cell Mol Life Sci* **76**: 2873‐83 [https://www.ncbi.nlm.nih.gov/pubmed/31123776]

Li J *et al*. (2019) Understanding histone H3 lysine 36 methylation and its deregulation in disease. *Cell Mol Life Sci* in press [https://www.ncbi.nlm.nih.gov/pubmed/31147750]

Shafabakhsh R *et al*. (2019) Role of histone modification and DNA methylation in signaling pathways involved in diabetic retinopathy. *J Cell Physiol.* **234**: 7839‐7846 [https://www.ncbi.nlm.nih.gov/pubmed/30515789]

### http://www.guidetopharmacology.org/GRAC/FamilyDisplayForward?familyId=783

#### Overview

Fatty acid‐binding proteins are low molecular weight (100‐130 aa) chaperones for long chain fatty acids, fatty acyl CoA esters, eicosanoids, retinols, retinoic acids and related metabolites and are usually regarded as being responsible for allowing the otherwise hydrophobic ligands to be mobile in aqueous media. These binding proteins may perform functions extracellularly (*e.g.* in plasma) or transport these agents; to the nucleus to interact with nuclear receptors (principally PPARs and retinoic acid receptors http://www.ncbi.nlm.nih.gov/pubmed/17882463?dopt=AbstractPlus]) or for interaction with metabolic enzymes. Although sequence homology is limited, crystallographic studies suggest conserved 3D structures across the group of binding proteins.

**Table:** 

Nomenclature	http://www.guidetopharmacology.org/GRAC/ObjectDisplayForward?objectId=2531	http://www.guidetopharmacology.org/GRAC/ObjectDisplayForward?objectId=2532	http://www.guidetopharmacology.org/GRAC/ObjectDisplayForward?objectId=2533	http://www.guidetopharmacology.org/GRAC/ObjectDisplayForward?objectId=2534	http://www.guidetopharmacology.org/GRAC/ObjectDisplayForward?objectId=2535
HGNC, UniProt	https://www.genenames.org/data/gene-symbol-report/#!/hgnc_id/HGNC:3555, http://www.uniprot.org/uniprot/P07148	https://www.genenames.org/data/gene-symbol-report/#!/hgnc_id/HGNC:3556, http://www.uniprot.org/uniprot/P12104	https://www.genenames.org/data/gene-symbol-report/#!/hgnc_id/HGNC:3557, http://www.uniprot.org/uniprot/P05413	https://www.genenames.org/data/gene-symbol-report/#!/hgnc_id/HGNC:3559, http://www.uniprot.org/uniprot/P15090	https://www.genenames.org/data/gene-symbol-report/#!/hgnc_id/HGNC:3560, http://www.uniprot.org/uniprot/Q01469
Rank order of potency	http://www.guidetopharmacology.org/GRAC/LigandDisplayForward?ligandId=3377, http://www.guidetopharmacology.org/GRAC/LigandDisplayForward?ligandId=1054 > http://www.guidetopharmacology.org/GRAC/LigandDisplayForward?ligandId=1055, http://www.guidetopharmacology.org/GRAC/LigandDisplayForward?ligandId=1052 > http://www.guidetopharmacology.org/GRAC/LigandDisplayForward?ligandId=2391, http://www.guidetopharmacology.org/GRAC/LigandDisplayForward?ligandId=1049 [http://www.ncbi.nlm.nih.gov/pubmed/7929039?dopt=AbstractPlus]	http://www.guidetopharmacology.org/GRAC/LigandDisplayForward?ligandId=3377 > http://www.guidetopharmacology.org/GRAC/LigandDisplayForward?ligandId=1055,http://www.guidetopharmacology.org/GRAC/LigandDisplayForward?ligandId=1054 > http://www.guidetopharmacology.org/GRAC/LigandDisplayForward?ligandId=1052 > http://www.guidetopharmacology.org/GRAC/LigandDisplayForward?ligandId=2391, http://www.guidetopharmacology.org/GRAC/LigandDisplayForward?ligandId=1049 [http://www.ncbi.nlm.nih.gov/pubmed/7929039?dopt=AbstractPlus]	http://www.guidetopharmacology.org/GRAC/LigandDisplayForward?ligandId=3377, http://www.guidetopharmacology.org/GRAC/LigandDisplayForward?ligandId=1054, http://www.guidetopharmacology.org/GRAC/LigandDisplayForward?ligandId=1055 > http://www.guidetopharmacology.org/GRAC/LigandDisplayForward?ligandId=1052, http://www.guidetopharmacology.org/GRAC/LigandDisplayForward?ligandId=1049, http://www.guidetopharmacology.org/GRAC/LigandDisplayForward?ligandId=2391 [http://www.ncbi.nlm.nih.gov/pubmed/7929039?dopt=AbstractPlus]	http://www.guidetopharmacology.org/GRAC/LigandDisplayForward?ligandId=1054, http://www.guidetopharmacology.org/GRAC/LigandDisplayForward?ligandId=1055, http://www.guidetopharmacology.org/GRAC/LigandDisplayForward?ligandId=3377, http://www.guidetopharmacology.org/GRAC/LigandDisplayForward?ligandId=1052 > http://www.guidetopharmacology.org/GRAC/LigandDisplayForward?ligandId=1049, http://www.guidetopharmacology.org/GRAC/LigandDisplayForward?ligandId=2391 [http://www.ncbi.nlm.nih.gov/pubmed/7929039?dopt=AbstractPlus]	–
Inhibitors	http://www.guidetopharmacology.org/GRAC/LigandDisplayForward?ligandId=7186 (p*K*_i_ 7.6) [http://www.ncbi.nlm.nih.gov/pubmed/18533710?dopt=AbstractPlus] – Rat, http://www.guidetopharmacology.org/GRAC/LigandDisplayForward?ligandId=2662 (p*K*_i_ 6.5) [http://www.ncbi.nlm.nih.gov/pubmed/18533710?dopt=AbstractPlus] – Rat, http://www.guidetopharmacology.org/GRAC/LigandDisplayForward?ligandId=6736 (p*K*_i_ 5.1) [http://www.ncbi.nlm.nih.gov/pubmed/19754198?dopt=AbstractPlus] – Mouse	–	–	–	http://www.guidetopharmacology.org/GRAC/LigandDisplayForward?ligandId=8797 (p*K*_i_ 8.7) [http://www.ncbi.nlm.nih.gov/pubmed/17502136?dopt=AbstractPlus]
Selective inhibitors	–	–	–	http://www.guidetopharmacology.org/GRAC/LigandDisplayForward?ligandId=8788 (p*K*_i_ >9) [http://www.ncbi.nlm.nih.gov/pubmed/21481589?dopt=AbstractPlus]	–
Comments	A broader substrate specificity than other FABPs, binding two fatty acids per protein [http://www.ncbi.nlm.nih.gov/pubmed/9054409?dopt=AbstractPlus].	Crystal structure of the rat FABP2 [http://www.ncbi.nlm.nih.gov/pubmed/2671390?dopt=AbstractPlus].	Crystal structure of the human FABP3 [http://www.ncbi.nlm.nih.gov/pubmed/7922029?dopt=AbstractPlus].	–	Crystal structure of the human FABP5 [http://www.ncbi.nlm.nih.gov/pubmed/10493790?dopt=AbstractPlus].

**Table:** 

Nomenclature	http://www.guidetopharmacology.org/GRAC/ObjectDisplayForward?objectId=2536	http://www.guidetopharmacology.org/GRAC/ObjectDisplayForward?objectId=2537	http://www.guidetopharmacology.org/GRAC/ObjectDisplayForward?objectId=2544	http://www.guidetopharmacology.org/GRAC/ObjectDisplayForward?objectId=2538	http://www.guidetopharmacology.org/GRAC/ObjectDisplayForward?objectId=2584
HGNC, UniProt	https://www.genenames.org/data/gene-symbol-report/#!/hgnc_id/HGNC:3561, http://www.uniprot.org/uniprot/P51161	https://www.genenames.org/data/gene-symbol-report/#!/hgnc_id/HGNC:3562, http://www.uniprot.org/uniprot/O15540	https://www.genenames.org/data/gene-symbol-report/#!/hgnc_id/HGNC:9117, http://www.uniprot.org/uniprot/P02689	https://www.genenames.org/data/gene-symbol-report/#!/hgnc_id/HGNC:3563, http://www.uniprot.org/uniprot/Q0Z7S8	https://www.genenames.org/data/gene-symbol-report/#!/hgnc_id/HGNC:34524, http://www.uniprot.org/uniprot/A6NFH5
Comments	Able to transport bile acids [http://www.ncbi.nlm.nih.gov/pubmed/23603607?dopt=AbstractPlus].	Crystal structure of the human FABP7 [http://www.ncbi.nlm.nih.gov/pubmed/10854433?dopt=AbstractPlus].	*In silico* modelling suggests that PMP2/FABP8 can bind both fatty acids and cholesterol [http://www.ncbi.nlm.nih.gov/pubmed/20421974?dopt=AbstractPlus].	–	–

**Table:** 

Nomenclature	http://www.guidetopharmacology.org/GRAC/ObjectDisplayForward?objectId=2546	http://www.guidetopharmacology.org/GRAC/ObjectDisplayForward?objectId=2547	http://www.guidetopharmacology.org/GRAC/ObjectDisplayForward?objectId=2548	http://www.guidetopharmacology.org/GRAC/ObjectDisplayForward?objectId=2549	http://www.guidetopharmacology.org/GRAC/ObjectDisplayForward?objectId=2550		http://www.guidetopharmacology.org/GRAC/ObjectDisplayForward?objectId=2551
HGNC, UniProt	https://www.genenames.org/data/gene-symbol-report/#!/hgnc_id/HGNC:9919, http://www.uniprot.org/uniprot/P09455	https://www.genenames.org/data/gene-symbol-report/#!/hgnc_id/HGNC:9920, http://www.uniprot.org/uniprot/P50120	https://www.genenames.org/data/gene-symbol-report/#!/hgnc_id/HGNC:9921, http://www.uniprot.org/uniprot/P10745	https://www.genenames.org/data/gene-symbol-report/#!/hgnc_id/HGNC:9922, http://www.uniprot.org/uniprot/P02753	https://www.genenames.org/data/gene-symbol-report/#!/hgnc_id/HGNC:15847, http://www.uniprot.org/uniprot/P82980		https://www.genenames.org/data/gene-symbol-report/#!/hgnc_id/HGNC:30316, http://www.uniprot.org/uniprot/Q96R05
Rank order of potency	–	http://www.guidetopharmacology.org/GRAC/LigandDisplayForward?ligandId=3377 > http://www.guidetopharmacology.org/GRAC/LigandDisplayForward?ligandId=1055, http://www.guidetopharmacology.org/GRAC/LigandDisplayForward?ligandId=1054, http://www.guidetopharmacology.org/GRAC/LigandDisplayForward?ligandId=1052, http://www.guidetopharmacology.org/GRAC/LigandDisplayForward?ligandId=1049, http://www.guidetopharmacology.org/GRAC/LigandDisplayForward?ligandId=2391 [http://www.ncbi.nlm.nih.gov/pubmed/10852718?dopt=AbstractPlus]	–	–	–	–	
Inhibitors	–	–	–	http://www.guidetopharmacology.org/GRAC/LigandDisplayForward?ligandId=8792 (pIC_50_ 7.8) [http://www.ncbi.nlm.nih.gov/pubmed/24835984?dopt=AbstractPlus]	–	–

**Table:** 

Nomenclature	http://www.guidetopharmacology.org/GRAC/ObjectDisplayForward?objectId=2545	http://www.guidetopharmacology.org/GRAC/ObjectDisplayForward?objectId=2529	http://www.guidetopharmacology.org/GRAC/ObjectDisplayForward?objectId=2530
HGNC, UniProt	https://www.genenames.org/data/gene-symbol-report/#!/hgnc_id/HGNC:10024, http://www.uniprot.org/uniprot/P12271	https://www.genenames.org/data/gene-symbol-report/#!/hgnc_id/HGNC:2338, http://www.uniprot.org/uniprot/P29762	https://www.genenames.org/data/gene-symbol-report/#!/hgnc_id/HGNC:2339, http://www.uniprot.org/uniprot/P29373
Rank order of potency	http://www.guidetopharmacology.org/GRAC/LigandDisplayForward?ligandId=6669, http://www.guidetopharmacology.org/GRAC/LigandDisplayForward?ligandId=6670 > http://www.guidetopharmacology.org/GRAC/LigandDisplayForward?ligandId=6673, http://www.guidetopharmacology.org/GRAC/LigandDisplayForward?ligandId=6671, http://www.guidetopharmacology.org/GRAC/LigandDisplayForward?ligandId=6672, http://www.guidetopharmacology.org/GRAC/LigandDisplayForward?ligandId=2350, http://www.guidetopharmacology.org/GRAC/LigandDisplayForward?ligandId=4053 [http://www.ncbi.nlm.nih.gov/pubmed/9541407?dopt=AbstractPlus]	http://www.guidetopharmacology.org/GRAC/LigandDisplayForward?ligandId=2644 > http://www.guidetopharmacology.org/GRAC/LigandDisplayForward?ligandId=2645 http://www.guidetopharmacology.org/GRAC/LigandDisplayForward?ligandId=3377 > http://www.guidetopharmacology.org/GRAC/LigandDisplayForward?ligandId=1055, http://www.guidetopharmacology.org/GRAC/LigandDisplayForward?ligandId=1054, http://www.guidetopharmacology.org/GRAC/LigandDisplayForward?ligandId=1052, http://www.guidetopharmacology.org/GRAC/LigandDisplayForward?ligandId=1049, http://www.guidetopharmacology.org/GRAC/LigandDisplayForward?ligandId=2391 [http://www.ncbi.nlm.nih.gov/pubmed/10852718?dopt=AbstractPlus]	–

#### Comments

Although not tested at all FABPs, http://www.guidetopharmacology.org/GRAC/LigandDisplayForward?ligandId=6735 exhibits high affinity for FABP4 (pIC50 8.8) compared to FABP3 or FABP5 (pIC50 <6.6) [http://www.ncbi.nlm.nih.gov/pubmed/17554340?dopt=AbstractPlus, http://www.ncbi.nlm.nih.gov/pubmed/17502136?dopt=AbstractPlus]. http://www.guidetopharmacology.org/GRAC/LigandDisplayForward?ligandId=6736 is reported to interfere with FABP4 action [http://www.ncbi.nlm.nih.gov/pubmed/19754198?dopt=AbstractPlus]. Ibuprofen displays some selectivity for FABP4 (pIC_50_ 5.5) relative to FABP3 (pIC_50_ 3.5) and FABP5 (pIC_50_ 3.8) [http://www.ncbi.nlm.nih.gov/pubmed/24248795?dopt=AbstractPlus]. Fenofibric acid displays some selectivity for FABP5 (pIC_50_ 5.5) relative to FABP3 (pIC_50_ 4.5) and FABP4 (pIC_50_ 4.6) [http://www.ncbi.nlm.nih.gov/pubmed/24248795?dopt=AbstractPlus]. Multiple pseudogenes for the FABPs have been identified in the human genome.

#### Further reading on Fatty acid‐binding proteins

Gajda AM *et al*. (2015) Enterocyte fatty acid‐binding proteins (FABPs): different functions of liver and intestinal FABPs in the intestine. *Prostaglandins Leukot. Essent. Fatty Acids* **93**: 9‐16 [https://www.ncbi.nlm.nih.gov/pubmed/25458898?dopt=AbstractPlus]

Glatz JF. (2015) Lipids and lipid binding proteins: a perfect match. *Prostaglandins Leukot Essent Fatty Acids* **93**: 45‐9 [https://www.ncbi.nlm.nih.gov/pubmed/25154384?dopt=AbstractPlus]

Hotamisligil GS *et al*. (2015) Metabolic functions of FABPs–mechanisms and therapeutic implications. *Nat Rev Endocrinol* **11**: 592‐605 [https://www.ncbi.nlm.nih.gov/pubmed/26260145?dopt=AbstractPlus]

Matsumata M *et al*. (2016) Fatty acid binding proteins and the nervous system: Their impact on mental conditions. *Neurosci. Res.* **102**: 47‐55 [https://www.ncbi.nlm.nih.gov/pubmed/25205626?dopt=AbstractPlus]

Osumi T *et al*. (2016) Heart lipid droplets and lipid droplet‐binding proteins: Biochemistry, physiology, and pathology. *Exp. Cell Res.* **340**: 198‐204 [https://www.ncbi.nlm.nih.gov/pubmed/26524506?dopt=AbstractPlus]

### http://www.guidetopharmacology.org/GRAC/FamilyDisplayForward?familyId=914

#### Overview

The canonical Notch signalling pathway has four type I transmembrane Notch receptors (Notch1‐4) and five ligands (DLL1, 2 and 3, and Jagged 1‐2). Each member of this highly conserved receptor family plays a unique role in cell‐fate determination during embryogenesis, differentiation, tissue patterning, proliferation and cell death [http://www.ncbi.nlm.nih.gov/pubmed/20971825?dopt=AbstractPlus]. As the Notch ligands are also membrane bound, cells have to be in close proximity for receptorligand interactions to occur. Cleavage of the intracellular domain (ICD) of activated Notch receptors by γ‐secretase is required for downstream signalling and Notch‐induced transcriptional modulation [http://www.ncbi.nlm.nih.gov/pubmed/10206645?dopt=AbstractPlus, http://www.ncbi.nlm.nih.gov/pubmed/16530044?dopt=AbstractPlus, http://www.ncbi.nlm.nih.gov/pubmed/9620803?dopt=AbstractPlus, http://www.ncbi.nlm.nih.gov/pubmed/16530045?dopt=AbstractPlus]. This is why γ‐secretase inhibitors can be used to downregulate Notch signalling and explains their anticancer action. One such small molecule is http://www.guidetopharmacology.org/GRAC/LigandDisplayForward?ligandId=7338 [http://www.ncbi.nlm.nih.gov/pubmed/19773430?dopt=AbstractPlus], although development of this compound has been terminated following an unsuccessful Phase II single agent clinical trial in metastatic colorectal cancer [http://www.ncbi.nlm.nih.gov/pubmed/22445247?dopt=AbstractPlus].

Aberrant Notch signalling is implicated in a number of human cancers [http://www.ncbi.nlm.nih.gov/pubmed/17344417?dopt=AbstractPlus, http://www.ncbi.nlm.nih.gov/pubmed/24651013?dopt=AbstractPlus, http://www.ncbi.nlm.nih.gov/pubmed/18079963?dopt=AbstractPlus, http://www.ncbi.nlm.nih.gov/pubmed/17173050?dopt=AbstractPlus], with http://www.guidetopharmacology.org/GRAC/LigandDisplayForward?ligandId=8451 and http://www.guidetopharmacology.org/GRAC/LigandDisplayForward?ligandId=8453 identified as antibody inhibitors of ligand:receptor binding [http://www.ncbi.nlm.nih.gov/pubmed/25388163?dopt=AbstractPlus].

**Table:** 

Nomenclature	http://www.guidetopharmacology.org/GRAC/ObjectDisplayForward?objectId=2861	http://www.guidetopharmacology.org/GRAC/ObjectDisplayForward?objectId=2859	http://www.guidetopharmacology.org/GRAC/ObjectDisplayForward?objectId=2860	http://www.guidetopharmacology.org/GRAC/ObjectDisplayForward?objectId=2862
HGNC, UniProt	https://www.genenames.org/data/gene-symbol-report/#!/hgnc_id/HGNC:7881, http://www.uniprot.org/uniprot/P46531	https://www.genenames.org/data/gene-symbol-report/#!/hgnc_id/HGNC:7882, http://www.uniprot.org/uniprot/Q04721	https://www.genenames.org/data/gene-symbol-report/#!/hgnc_id/HGNC:7883, http://www.uniprot.org/uniprot/Q9UM47	https://www.genenames.org/data/gene-symbol-report/#!/hgnc_id/HGNC:7884, http://www.uniprot.org/uniprot/Q99466
Comments	Various types of activating and inactivating *NOTCH1* mutations have been reported to be associated with human diseases, for example: aortic valve disease [http://www.ncbi.nlm.nih.gov/pubmed/16025100?dopt=AbstractPlus, http://www.ncbi.nlm.nih.gov/pubmed/18593716?dopt=AbstractPlus], Adams‐Oliver syndrome 5 [http://www.ncbi.nlm.nih.gov/pubmed/25132448?dopt=AbstractPlus], T‐cell acute lymphoblastic leukemia (T‐ALL) [http://www.ncbi.nlm.nih.gov/pubmed/15472075?dopt=AbstractPlus], chronic lymphocytic leukemia (CLL) [http://www.ncbi.nlm.nih.gov/pubmed/21642962?dopt=AbstractPlus] and head and neck squamous cell carcinoma [http://www.ncbi.nlm.nih.gov/pubmed/21798897?dopt=AbstractPlus, http://www.ncbi.nlm.nih.gov/pubmed/21798893?dopt=AbstractPlus].	–	–	Notch receptor 4 is a potential therapeutic molecular target for triple‐negative breast cancer [http://www.ncbi.nlm.nih.gov/pubmed/25993190?dopt=AbstractPlus, http://www.ncbi.nlm.nih.gov/pubmed/24403446?dopt=AbstractPlus].

#### Further reading on Notch receptors

Arumugam TV *et al*. (2018) Notch signaling and neuronal death in stroke. *Prog. Neurobiol.* **165‐167**: 103‐116 [https://www.ncbi.nlm.nih.gov/pubmed/29574014?dopt=AbstractPlus]

Borggrefe T *et al*. (2016) The Notch intracellular domain integrates signals from Wnt, Hedgehog, TGFβ/BMP and hypoxia pathways. *Biochim. Biophys. Acta* **1863**: 303‐13 [https://www.ncbi.nlm.nih.gov/pubmed/26592459?dopt=AbstractPlus]

Palmer WH *et al*. (2015) Ligand‐Independent Mechanisms of Notch Activity. *Trends Cell Biol.* **25**: 697‐707 [https://www.ncbi.nlm.nih.gov/pubmed/26437585?dopt=AbstractPlus]

Previs RA *et al*. (2015) Molecular pathways: translational and therapeutic implications of the Notch signaling pathway in cancer. *Clin. Cancer Res.* **21**: 955‐61 [https://www.ncbi.nlm.nih.gov/pubmed/25388163?dopt=AbstractPlus]

Takebe N *et al*. (2015) Targeting Notch, Hedgehog, and Wnt pathways in cancer stem cells: clinical update. *Nat Rev Clin Oncol* **12**: 445‐64 [https://www.ncbi.nlm.nih.gov/pubmed/25850553?dopt=AbstractPlus]

### http://www.guidetopharmacology.org/GRAC/FamilyDisplayForward?familyId=891

#### Overview

Regulators of G protein signalling (RGS) proteins display a common RGS domain that interacts with the GTP‐bound Gα subunits of heterotrimeric G proteins, enhancing GTP hydrolysis by stabilising the transition state [http://www.ncbi.nlm.nih.gov/pubmed/8756726?dopt=AbstractPlus, http://www.ncbi.nlm.nih.gov/pubmed/9108480?dopt=AbstractPlus, http://www.ncbi.nlm.nih.gov/pubmed/9417641?dopt=AbstractPlus], leading to a termination of GPCR signalling. Interactions through protein: protein interactions of many RGS proteins have been identified for targets other than heteromeric G proteins. Sequence analysis of the 20 RGS proteins suggests four families of RGS: RZ, R4, R7 and R12 families. Many of these proteins have been identified to have effects other than through targetting G proteins. Included here is RGS4 for which a number of pharmacological inhibitors have been described.

### http://www.guidetopharmacology.org/GRAC/FamilyDisplayForward?familyId=892

#### Overview

The RZ family of RGS proteins is less well characterized than the other families [http://www.ncbi.nlm.nih.gov/pubmed/16765607?dopt=AbstractPlus]. It consists of RGS17 (also known as RGSZ2), RGS19 (also known as GAIP) and RGS20 (with several splice variants including RGSZ1 and Ret‐RGS). All members contain an N‐terminal cysteine string motif [http://www.ncbi.nlm.nih.gov/pubmed/17183362?dopt=AbstractPlus] which is a site of palmitoylation and could serve functions in membrane targeting, protein stability or aid protein‐protein interactions [http://www.ncbi.nlm.nih.gov/pubmed/17126529?dopt=AbstractPlus]. However, the function in the case of RZ family RGS proteins is not yet fully understood. Members of the RZ family of RGS proteins are the only RGS proteins that have selective GTPase activating‐protein (GAP) activity for Gα_z_, a function that resulted in the name of the family [http://www.ncbi.nlm.nih.gov/pubmed/9748279?dopt=AbstractPlus, http://www.ncbi.nlm.nih.gov/pubmed/15096504?dopt=AbstractPlus, http://www.ncbi.nlm.nih.gov/pubmed/9748280?dopt=AbstractPlus, http://www.ncbi.nlm.nih.gov/pubmed/1347957?dopt=AbstractPlus]. However, the members of the RZ family are able to also GAP Gα_i/o_ members with varying selectivity.

**Table:** 

Nomenclature	http://www.guidetopharmacology.org/GRAC/ObjectDisplayForward?objectId=2801	http://www.guidetopharmacology.org/GRAC/ObjectDisplayForward?objectId=2802	http://www.guidetopharmacology.org/GRAC/ObjectDisplayForward?objectId=2803
Common abbreviation	RGS17	RGS19	RGS20
HGNC, UniProt	https://www.genenames.org/data/gene-symbol-report/#!/hgnc_id/HGNC:14088, http://www.uniprot.org/uniprot/Q9UGC6	https://www.genenames.org/data/gene-symbol-report/#!/hgnc_id/HGNC:13735, http://www.uniprot.org/uniprot/P49795	https://www.genenames.org/data/gene-symbol-report/#!/hgnc_id/HGNC:14600, http://www.uniprot.org/uniprot/O76081

### http://www.guidetopharmacology.org/GRAC/FamilyDisplayForward?familyId=893

#### Overview

This is the largest family of RGS proteins.

**Table:** 

Nomenclature	http://www.guidetopharmacology.org/GRAC/ObjectDisplayForward?objectId=2804	http://www.guidetopharmacology.org/GRAC/ObjectDisplayForward?objectId=2808	http://www.guidetopharmacology.org/GRAC/ObjectDisplayForward?objectId=2810	http://www.guidetopharmacology.org/GRAC/ObjectDisplayForward?objectId=2811
Common abbreviation	RGS1	RGS2	RGS3	RGS4
HGNC, UniProt	https://www.genenames.org/data/gene-symbol-report/#!/hgnc_id/HGNC:9991, http://www.uniprot.org/uniprot/Q08116	https://www.genenames.org/data/gene-symbol-report/#!/hgnc_id/HGNC:9998, http://www.uniprot.org/uniprot/P41220	https://www.genenames.org/data/gene-symbol-report/#!/hgnc_id/HGNC:9999, http://www.uniprot.org/uniprot/P49796	https://www.genenames.org/data/gene-symbol-report/#!/hgnc_id/HGNC:10000, http://www.uniprot.org/uniprot/P49798
Selective inhibitors	–	–	–	http://www.guidetopharmacology.org/GRAC/LigandDisplayForward?ligandId=8033 (pIC_50_ 7.8) [http://www.ncbi.nlm.nih.gov/pubmed/22368763?dopt=AbstractPlus], http://www.guidetopharmacology.org/GRAC/LigandDisplayForward?ligandId=7784 (pIC_50_ 7.5) [http://www.ncbi.nlm.nih.gov/pubmed/21329361?dopt=AbstractPlus], http://www.guidetopharmacology.org/GRAC/LigandDisplayForward?ligandId=8034 (pIC_50_ 7.3) [http://www.ncbi.nlm.nih.gov/pubmed/22368763?dopt=AbstractPlus]

**Table:** 

Nomenclature	http://www.guidetopharmacology.org/GRAC/ObjectDisplayForward?objectId=2812	http://www.guidetopharmacology.org/GRAC/ObjectDisplayForward?objectId=2813	http://www.guidetopharmacology.org/GRAC/ObjectDisplayForward?objectId=2805	http://www.guidetopharmacology.org/GRAC/ObjectDisplayForward?objectId=2806	http://www.guidetopharmacology.org/GRAC/ObjectDisplayForward?objectId=2807	http://www.guidetopharmacology.org/GRAC/ObjectDisplayForward?objectId=2809
Common abbreviation	RGS5	RGS8	RGS13	RGS16	RGS18	RGS21
HGNC, UniProt	https://www.genenames.org/data/gene-symbol-report/#!/hgnc_id/HGNC:10001, http://www.uniprot.org/uniprot/O15539	https://www.genenames.org/data/gene-symbol-report/#!/hgnc_id/HGNC:16810, http://www.uniprot.org/uniprot/P57771	https://www.genenames.org/data/gene-symbol-report/#!/hgnc_id/HGNC:9995, http://www.uniprot.org/uniprot/O14921	https://www.genenames.org/data/gene-symbol-report/#!/hgnc_id/HGNC:9997, http://www.uniprot.org/uniprot/O15492	https://www.genenames.org/data/gene-symbol-report/#!/hgnc_id/HGNC:14261, http://www.uniprot.org/uniprot/Q9NS28	https://www.genenames.org/data/gene-symbol-report/#!/hgnc_id/HGNC:26839, http://www.uniprot.org/uniprot/Q2M5E4

### http://www.guidetopharmacology.org/GRAC/FamilyDisplayForward?familyId=894

#### Overview

This family of RGS proteins shows some selectivity for Gai/o proteins.

**Table:** 

Nomenclature	http://www.guidetopharmacology.org/GRAC/ObjectDisplayForward?objectId=2815	http://www.guidetopharmacology.org/GRAC/ObjectDisplayForward?objectId=2816	http://www.guidetopharmacology.org/GRAC/ObjectDisplayForward?objectId=2817	http://www.guidetopharmacology.org/GRAC/ObjectDisplayForward?objectId=2814
Common abbreviation	RGS6	RGS7	RGS9	RGS11
HGNC, UniProt	https://www.genenames.org/data/gene-symbol-report/#!/hgnc_id/HGNC:10002, http://www.uniprot.org/uniprot/P49758	https://www.genenames.org/data/gene-symbol-report/#!/hgnc_id/HGNC:10003, http://www.uniprot.org/uniprot/P49802	https://www.genenames.org/data/gene-symbol-report/#!/hgnc_id/HGNC:10004, http://www.uniprot.org/uniprot/O75916	https://www.genenames.org/data/gene-symbol-report/#!/hgnc_id/HGNC:9993, http://www.uniprot.org/uniprot/O94810

### http://www.guidetopharmacology.org/GRAC/FamilyDisplayForward?familyId=895

#### Overview

The R12 family consists of RGS10, 12 and 14. RGS12 and 14 are large proteins with additional domains that can participate in protein‐protein interactions and other functions. In contrast, RGS10 is a small protein consisting of the RGS domain and small N‐ and C‐termini, similar to members of the R4 family. However, sequence homology of the RGS10 RGS domain clearly places it in the R12 family [http://www.ncbi.nlm.nih.gov/pubmed/26123306?dopt=AbstractPlus]. The Gα_i/o_‐Loco (GoLoco) motif in RGS12 and 14 has GDI activity (for Guanine nucleotide Dissociation Inhibitor) towards Gα_i1_, Gα_i2_ and Gα_i3_ [http://www.ncbi.nlm.nih.gov/pubmed/11387333?dopt=AbstractPlus, http://www.ncbi.nlm.nih.gov/pubmed/15951850?dopt=AbstractPlus]. Through this activity RGS12 and RGS14 can inhibit G protein signaling both by accelerating GTP hydrolysis and by preventing G protein activation. Splice variants of RGS12 and RGS14 also contain membrane targeting and protein‐protein interaction domains [http://www.ncbi.nlm.nih.gov/pubmed/11130074?dopt=AbstractPlus, http://www.ncbi.nlm.nih.gov/pubmed/11771424?dopt=AbstractPlus, http://www.ncbi.nlm.nih.gov/pubmed/9651375?dopt=AbstractPlus].

**Table:** 

Nomenclature	http://www.guidetopharmacology.org/GRAC/ObjectDisplayForward?objectId=2818	http://www.guidetopharmacology.org/GRAC/ObjectDisplayForward?objectId=2819	http://www.guidetopharmacology.org/GRAC/ObjectDisplayForward?objectId=2820
Common abbreviation	RGS10	RGS12	RGS14
HGNC, UniProt	https://www.genenames.org/data/gene-symbol-report/#!/hgnc_id/HGNC:9992, http://www.uniprot.org/uniprot/O43665	https://www.genenames.org/data/gene-symbol-report/#!/hgnc_id/HGNC:9994, http://www.uniprot.org/uniprot/O14924	https://www.genenames.org/data/gene-symbol-report/#!/hgnc_id/HGNC:9996, http://www.uniprot.org/uniprot/O43566

#### Further reading on Regulators of G protein Signaling (RGS) proteins

Alqinyah M *et al*. (2018) Regulating the regulators: Epigenetic, transcriptional, and post‐translational regulation of RGS proteins. *Cell. Signal.* **42**: 77‐87 [https://www.ncbi.nlm.nih.gov/pubmed/29042285?dopt=AbstractPlus]

Neubig RR *et al*. (2002) Regulators of G‐protein signalling as new central nervous system drug targets. *Nat Rev Drug Discov* **1**: 187‐97 [https://www.ncbi.nlm.nih.gov/pubmed/12120503?dopt=AbstractPlus]

Sethakorn N *et al*. (2010) Non‐canonical functions of RGS proteins. *Cell. Signal.* **22**: 1274‐81 [https://www.ncbi.nlm.nih.gov/pubmed/20363320?dopt=AbstractPlus]

Sjögren B. (2017) The evolution of regulators of G protein signalling proteins as drug targets ‐ 20 years in the making: IUPHAR Review 21. *Br. J. Pharmacol.* **174**: 427‐437 [https://www.ncbi.nlm.nih.gov/pubmed/28098342?dopt=AbstractPlus]

Sjögren B *et al*. (2010) Thinking outside of the ‘RGS box’: new approaches to therapeutic targeting of regulators of G protein signaling. *Mol. Pharmacol.* **78**: 550‐7 [https://www.ncbi.nlm.nih.gov/pubmed/20664002?dopt=AbstractPlus]

### http://www.guidetopharmacology.org/GRAC/FamilyDisplayForward?familyId=785

#### Overview

Although termed ’receptors’, the evidence for coupling through conventional signalling pathways is lacking. Initially described as a subtype of opioid receptors, there is only a modest pharmacological overlap and no structural convergence with the G protein‐coupled receptors; the crystal structure of the sigma1 receptor [http://www.ncbi.nlm.nih.gov/pubmed/27042935?dopt=AbstractPlus] suggests a trimeric structure of a single short transmembrane domain traversing the endoplasmic reticulum membrane, with the bulk of the protein facing the cytosol. A wide range of compounds, ranging from psychoactive agents to antihistamines, have been observed to bind to these sites.

**Table:** 

Nomenclature	http://www.guidetopharmacology.org/GRAC/ObjectDisplayForward?objectId=2552	http://www.guidetopharmacology.org/GRAC/ObjectDisplayForward?objectId=2553
HGNC, UniProt	https://www.genenames.org/data/gene-symbol-report/#!/hgnc_id/HGNC:8157, http://www.uniprot.org/uniprot/Q99720	https://www.genenames.org/data/gene-symbol-report/#!/hgnc_id/HGNC:28106, http://www.uniprot.org/uniprot/Q5BJF2
Agonists	–	http://www.guidetopharmacology.org/GRAC/LigandDisplayForward?ligandId=6684 [http://www.ncbi.nlm.nih.gov/pubmed/16463398?dopt=AbstractPlus] – Guinea pig
Selective agonists	http://www.guidetopharmacology.org/GRAC/LigandDisplayForward?ligandId=6678 [http://www.ncbi.nlm.nih.gov/pubmed/1658302?dopt=AbstractPlus], (http://www.guidetopharmacology.org/GRAC/LigandDisplayForward?ligandId=6677	–
Antagonists	–	http://www.guidetopharmacology.org/GRAC/LigandDisplayForward?ligandId=6682 (pIC_50_ 7.2) [http://www.ncbi.nlm.nih.gov/pubmed/10096443?dopt=AbstractPlus]
Selective antagonists	http://www.guidetopharmacology.org/GRAC/LigandDisplayForward?ligandId=6679 (pIC_50_ 8.4) [http://www.ncbi.nlm.nih.gov/pubmed/7901723?dopt=AbstractPlus], http://www.guidetopharmacology.org/GRAC/LigandDisplayForward?ligandId=6680 (pIC_50_ 7.4) [http://www.ncbi.nlm.nih.gov/pubmed/8566098?dopt=AbstractPlus]	–
Labelled ligands	[http://www.guidetopharmacology.org/GRAC/LigandDisplayForward?ligandId=6683 (Agonist)	[http://www.guidetopharmacology.org/GRAC/LigandDisplayForward?ligandId=6685 (Agonist)
Comments	–	The sigma2 receptor has been reported to be TMEM97 [http://www.ncbi.nlm.nih.gov/pubmed/28559337?dopt=AbstractPlus], a 4TM protein partner of NPC1, the Niemann‐Pick C1 protein, a 13TM cholesterol‐binding protein.

#### Comments

http://www.guidetopharmacology.org/GRAC/LigandDisplayForward?ligandId=1606 also shows activity at opioid receptors. The sigma2 receptor has recently been reported to be http://www.guidetopharmacology.org/GRAC/ObjectDisplayForward?objectId=2553 [http://www.ncbi.nlm.nih.gov/pubmed/28559337?dopt=AbstractPlus], a 4TM protein partner of NPC1, the Niemann‐Pick C1 protein, a 13TM cholesterol‐binding protein.

#### Further reading on Sigma receptors

Chu UB *et al*. (2016) Biochemical Pharmacology of the Sigma‐1 Receptor. *Mol. Pharmacol.* **89**: 142‐53 [https://www.ncbi.nlm.nih.gov/pubmed/26560551?dopt=AbstractPlus]

Gris G *et al*. (2015) Sigma‐1 receptor and inflammatory pain. *Inflamm. Res.* **64**: 377‐81 [https://www.ncbi.nlm.nih.gov/pubmed/25902777?dopt=AbstractPlus]

Rousseaux CG *et al*. (2016) Sigma receptors [*σ*Rs]: biology in normal and diseased states. *J. Recept. Signal Transduct. Res.* **36**: 327‐388 [https://www.ncbi.nlm.nih.gov/pubmed/26056947?dopt=AbstractPlus]

Sambo DO *et al*. (2018) The sigma‐1 receptor as a regulator of dopamine neurotransmission: A potential therapeutic target for methamphetamine addiction. *Pharmacol Ther* **186**: 152‐167 [https://www.ncbi.nlm.nih.gov/pubmed/29360540]

Su TP *et al*. (2016) The Sigma‐1 Receptor as a Pluripotent Modulator in Living Systems. *Trends Pharmacol. Sci.* **37**: 262‐278 [https://www.ncbi.nlm.nih.gov/pubmed/26869505?dopt=AbstractPlus]

Vavers E *et al*. (2019) Allosteric Modulators of Sigma‐1 Receptor: A Review. *Front Pharmacol* **10**: 223 [https://www.ncbi.nlm.nih.gov/pubmed/30941035]

### http://www.guidetopharmacology.org/GRAC/FamilyDisplayForward?familyId=858

#### Overview

Tubulins are a family of intracellular proteins most commonly associated with microtubules, part of the cytoskeleton. They are exploited for therapeutic gain in cancer chemotherapy as targets for agents derived from a variety of natural products: taxanes, colchicine and vinca alkaloids. These are thought to act primarily through β‐tubulin, thereby interfering with the normal processes of tubulin polymer formation and disassembly.

**Table:** 

Nomenclature	http://www.guidetopharmacology.org/GRAC/ObjectDisplayForward?objectId=2638	http://www.guidetopharmacology.org/GRAC/ObjectDisplayForward?objectId=2639	http://www.guidetopharmacology.org/GRAC/ObjectDisplayForward?objectId=2640	http://www.guidetopharmacology.org/GRAC/ObjectDisplayForward?objectId=2752	http://www.guidetopharmacology.org/GRAC/ObjectDisplayForward?objectId=2641	http://www.guidetopharmacology.org/GRAC/ObjectDisplayForward?objectId=2753
HGNC, UniProt	https://www.genenames.org/data/gene-symbol-report/#!/hgnc_id/HGNC:20766, http://www.uniprot.org/uniprot/Q71U36	https://www.genenames.org/data/gene-symbol-report/#!/hgnc_id/HGNC:12407, http://www.uniprot.org/uniprot/P68366	https://www.genenames.org/data/gene-symbol-report/#!/hgnc_id/HGNC:20778, http://www.uniprot.org/uniprot/P07437	https://www.genenames.org/data/gene-symbol-report/#!/hgnc_id/HGNC:20772, http://www.uniprot.org/uniprot/Q13509	https://www.genenames.org/data/gene-symbol-report/#!/hgnc_id/HGNC:20771, http://www.uniprot.org/uniprot/P68371	https://www.genenames.org/data/gene-symbol-report/#!/hgnc_id/HGNC:20773, http://www.uniprot.org/uniprot/Q3ZCM7
Inhibitors	–	–	http://www.guidetopharmacology.org/GRAC/LigandDisplayForward?ligandId=6851 (pIC_50_ 9), http://www.guidetopharmacology.org/GRAC/LigandDisplayForward?ligandId=6813 (pIC_50_ 8.2) [http://www.ncbi.nlm.nih.gov/pubmed/21324687?dopt=AbstractPlus], http://www.guidetopharmacology.org/GRAC/LigandDisplayForward?ligandId=2770 (Mitotic cell cycle arrest in A431 cells) (pEC_50_ 8.1) [http://www.ncbi.nlm.nih.gov/pubmed/16377187?dopt=AbstractPlus], http://www.guidetopharmacology.org/GRAC/LigandDisplayForward?ligandId=2367 (pIC_50_ 8) [http://www.ncbi.nlm.nih.gov/pubmed/16504507?dopt=AbstractPlus], http://www.guidetopharmacology.org/GRAC/LigandDisplayForward?ligandId=6798, http://www.guidetopharmacology.org/GRAC/LigandDisplayForward?ligandId=6809, http://www.guidetopharmacology.org/GRAC/LigandDisplayForward?ligandId=6824, http://www.guidetopharmacology.org/GRAC/LigandDisplayForward?ligandId=6785	http://www.guidetopharmacology.org/GRAC/LigandDisplayForward?ligandId=8854 (pIC_50_ 8.2) [http://www.ncbi.nlm.nih.gov/pubmed/23895532?dopt=AbstractPlus]	–	–

#### Further reading on Tubulins

Arnst KE *et al*. (2019) Current advances of tubulin inhibitors as dual acting small molecules for cancer therapy. *Med Res Rev* **39**: 1398‐1426 [https://www.ncbi.nlm.nih.gov/pubmed/30746734]

Eshun‐Wilson L. (2019) Effects of alpha‐tubulin acetylation on microtubule structure and stability. *Proc Natl Acad Sci U S A* **116**: 10366‐10371 [https://www.ncbi.nlm.nih.gov/pubmed/31072936]

Gadadhar S *et al*. (2017) The tubulin code at a glance. *J. Cell. Sci.* **130**: 1347‐1353 [https://www.ncbi.nlm.nih.gov/pubmed/28325758?dopt=AbstractPlus]

Magiera MM *et al*. (2018) Tubulin Posttranslational Modifications and Emerging Links to Human Disease. *Cell* **173**: 1323‐1327 [https://www.ncbi.nlm.nih.gov/pubmed/29856952]

Penna LS *et al*. (2017) Anti‐mitotic agents: Are they emerging molecules for cancer treatment? *Pharmacol. Ther.* **173**: 67‐82 [https://www.ncbi.nlm.nih.gov/pubmed/28174095?dopt=AbstractPlus]
